# Assessment of Post-COVID-19 Functional Status and Complications Among Survivors at a Tertiary Healthcare Center in Bangladesh

**DOI:** 10.7759/cureus.82866

**Published:** 2025-04-23

**Authors:** Mohammad Tariqul Islam, Afsana Mahjabin, Md. Mahbubul Islam, Anika Tasnim, Md. Rashid Al-Mahmood, Moshiur R Khasru, A.K.M. Salek, Taslim Uddin

**Affiliations:** 1 Physical Medicine and Rehabilitation, Bangabandhu Sheikh Mujib Medical University, Dhaka, BGD; 2 Community Medicine, Monno Medical College, Manikgonj, BGD; 3 Physical Medicine and Rehabilitation, Manikganj Sadar Hospital, Manikganj, BGD; 4 Public Health and Informatics, Bangabandhu Sheikh Mujib Medical University, Dhaka, BGD; 5 Physical Medicine and Rehabilitation, Bangladesh Medical College, Dhaka, BGD; 6 Physical Medicine and Rehabilitation, Northern International Medical College, Dhaka, BGD

**Keywords:** bangladesh, covid-19, functional status, healthcare, post-covid complications

## Abstract

Background and objectives

COVID-19 has caused widespread multisystem impairment, leaving survivors with significant post-COVID complications. Survivors have faced ongoing difficulties as a result of post-COVID complications.

This study aimed to assess the post-COVID functional status and complications among COVID-19 survivors within a tertiary healthcare center in Bangladesh.

Methodology

In this observational study, 244 patients were selected based on predefined inclusion and exclusion criteria from the post-COVID-19 follow-up clinic of the Department of Medicine at Bangabandhu Sheikh Mujib Medical University (BSMMU).

Results

COVID-19 functional status was assessed using the Post-COVID-19 Functional Status Scale (PCFS). Most of the participants belonged to grade 1, whereas the fewest belonged to grade 4. Fatigue (190, 77.9%), sleep disturbances (126, 51.6%), anxiety (102, 41.8%), and breathing difficulties (98, 40.2%) were the most prevalent complications. Sleep disturbances and breathing difficulties were notably associated with most grades of functional status. Sleep disturbances showed statistical significance with all grades except grade 4 (*P *≤ 0.05), while breathing difficulties were significantly associated with all grades except grade 2 (*P *≤ 0.05).

Conclusions

The results of this study shed light on the long-term consequences of COVID-19 in a Bangladeshi context and underscore the importance of continued care and support for survivors. Timely interventions and rehabilitation services can play a crucial role in improving the overall quality of life for COVID-19 survivors in Bangladesh and beyond.

## Introduction

The COVID-19 pandemic, caused by SARS-CoV-2, has led to significant morbidity and mortality worldwide. While the acute phase of the infection has been extensively studied, the long-term health consequences among survivors are becoming increasingly recognized. Post-COVID-19 syndrome refers to the persistence of symptoms even after the clearance of the virus and the development of new symptoms or worsening of pre-existing chronic conditions within a month of recovering from COVID-19 [1].

COVID-19 can present in various ways, ranging from no symptoms to severe and fatal cases [2]. Severe infections can lead to complications such as pneumonia, acute respiratory distress syndrome (ARDS), sepsis, organ failure, thrombosis, myocarditis, acute myocardial infarction, acute kidney injury, and secondary viral and bacterial infections. COVID-19-associated deaths are often the result of pneumonia and hyperinflammation, which are linked to a condition called cytokine storm syndrome [3-5]. Since the start of the COVID-19 pandemic, there has been a significant number of deaths and growing reports of long-term health issues worldwide [6,7]. While many individuals have recovered from the disease, there have been documented cases of ongoing functional and psychological problems [8]. The long-term functional status of COVID-19 survivors has not been extensively studied or documented in the Bangladeshi context. Even though individuals may be classified as recovered, they may still experience disabling consequences and could benefit from multidisciplinary rehabilitation to restore function in all aspects of life. Common long-term complaints among COVID-19 survivors include persistent pain (such as muscle pain, joint pain, and headaches), as well as symptoms like fatigue, anxiety, depression, memory and concentration issues, and sleep disorders [3,5,9].

In Bangladesh, the burden of COVID-19 has been substantial, with millions of confirmed cases and many recoveries. However, the long-term effects of COVID-19 on survivors in this area are still not well understood. It is essential to study the post-COVID functional status and complications to create specific healthcare strategies and rehabilitation programs that can effectively support these individuals. Limited research has been conducted on the post-COVID functional status and complications in the Bangladeshi population, despite the growing recognition of long COVID as a global health challenge. Understanding the prevalence and types of post-COVID complications is crucial for developing targeted healthcare strategies and rehabilitation programs to address the unique needs of survivors in low- and middle-income countries like Bangladesh.

This study aims to assess the post-COVID functional status and complications among COVID-19 survivors in a tertiary healthcare center in Bangladesh. Specifically, it seeks to assess the severity and types of functional limitations experienced by survivors and determine the distribution of their functional status and post-COVID-19 complications.

By evaluating the prevalence of long-term complications and their impact on daily functioning, this research seeks to provide valuable insights into the rehabilitation needs of COVID-19 survivors. Furthermore, it aims to inform healthcare providers and policymakers about the long-term healthcare requirements of this population, ultimately contributing to improved patient outcomes and quality of life. The findings of this study will also contribute to the growing body of evidence on long COVID, particularly in resource-limited settings, and highlight the importance of continued care and support for COVID-19 survivors.

## Materials and methods

Design

This was a cross-sectional study. It adhered to the Declaration of Helsinki (2013 revision) and did not involve any invasive procedures.

Clinical setting

The study was conducted in the Department of Physical Medicine & Rehabilitation at Bangabandhu Sheikh Mujib Medical University (BSMMU) from November 2020 to October 2021. Interviews took place in the post-COVID-19 follow-up clinic, with adherence to safety protocols, including mask-wearing and maintaining a six-foot distance.

Population and sample

Patients were selected from the post-COVID-19 follow-up clinic under the Department of Internal Medicine at BSMMU. The selection process was based on consecutive and convenience sampling. All patients who met the inclusion criteria and visited the clinic during the study period were approached for participation. Twice a week, for three months, every alternate patient was invited to participate. Eligibility was assessed based on age, confirmed past COVID-19 infection via reverse transcription polymerase chain reaction (RT-PCR), and the willingness to provide informed consent. Patients with a known history of psychiatric illnesses were excluded to avoid confounding psychological variables.

The sample size for this study was determined by using the formula *n* = *z*^2^*pq*/*d*^2^ = 382, where *n* is the sample size, *z* = 1.96 at 95% confidence level, *p*= prevalence of fatigue as post-COVID-19 complications, 53% [10], *q* = 1 - *p*, and *d *refers to the margin of error = 5%. But due to time and resource constraints, we included 244 patients in the study.

The study involved 244 patients selected based on specific inclusion and exclusion criteria from the post-COVID-19 follow-up clinic. Patients visited the post-COVID-19 follow-up clinic either two weeks after discharge from the hospital (for admitted patients) or two weeks after receiving a negative RT-PCR result (for non-admitted patients).

This two-week window was chosen based on clinical recommendations that patients should be symptomatically stable and non-infectious by this time, allowing a safer and more standardized point of evaluation for post-COVID complications.

Participants aged 18 to 90 years who tested positive for COVID-19 via RT-PCR and gave written consent were included. Individuals with a history of major depressive illness, schizophrenia, mental illness, or those on psychiatric medication were excluded. Patients were recruited either for two weeks post-hospital discharge or two weeks after a negative RT-PCR test if not hospitalized. During the interviews, participants were specifically asked about any history of reinfection. If a reinfection had occurred, the timeline and symptom details were recorded separately. However, such cases were minimal and did not significantly impact the core study objectives. Where reinfection was reported, it was documented but excluded from the main statistical analysis to maintain homogeneity in evaluating complications after a single infection. All patients were recruited either two weeks after discharge from the hospital or, for those not hospitalized, two weeks after obtaining a negative RT-PCR test result.

Data collection

Data collection involved face-to-face interviews conducted by trained research assistants using a semi-structured questionnaire. Participants provided information on sociodemographic variables, personal habits, duration of COVID-19 illness, experienced symptoms, co-morbidities, post-COVID-19 complications, and quality of life as assessed by the Post-COVID-19 Functional Status (PCFS) scale. The PCFS is a widely recognized tool for assessing functional limitations in COVID-19 patients and consists of five grades, ranging from 0 (no functional limitations) to 4 (severe functional limitations) (Appendix). Grade 0 indicates the complete absence of symptoms, with individuals able to perform all activities they could before contracting COVID-19. Grade 1 signifies no symptoms at rest but restrictions in activities requiring strenuous physical effort or intense mental focus. Grade 2 indicates the presence of symptoms at rest, though individuals can still manage self-care. Grade 3 reflects the need for some assistance with daily tasks such as cooking, cleaning, or shopping. Grade 4 denotes the need for assistance with basic activities of daily living, such as dressing or bathing [8]. Face-to-face interviews lasted less than 30 minutes, with participants assured of their right to withdraw anytime without explanation. No incentives were provided for participation. Our previous study provided a detailed methodology [11].

Statistical analysis

Statistical analyses were performed using SPSS version 26 (IBM Corp., Armonk, NY). A *P*-value ≤ 0.05 was considered indicative of statistical significance. The statistical analysis was performed using descriptive and inferential methods to evaluate the post-COVID-19 functional status and complications. Frequency distributions and percentages were calculated for demographic characteristics, comorbidities, drug history, and post-COVID-19 complications. The results are presented as counts (*n*) and percentages (%). Chi-square tests were conducted to assess the association between categorical variables, such as the presence of post-COVID-19 complications (e.g., fatigue, sleep disturbance, anxiety) and functional status across the PCFS grades.

Ethical permission

The study obtained ethical approval from BSMMU's Institutional Review Board (IRB) (memo no BSMMU/2020/9236, approved on October 19, 2020).

## Results

Among the 244 participants, 140 (57.4%) were male, with the majority falling within the 20-35 years age group (*n* = 92, 37.7%). Most participants had an education level above secondary school (*n* = 82, 82%) and were employed as service holders (*n* = 138, 56.6%). Most of them (*n *= 164, 67.2%) belonged to nuclear families. Hypertension, diabetes, and bronchial asthma were the most common comorbidities among the participants, reported by 92 (69.7%), 64 (48.5%), and 36 (27.3%) individuals, respectively. Correspondingly, antihypertensive and anti-diabetic medications were the most commonly used drugs, taken by 86 (71.7%) and 62 (51.7%) participants, respectively. Among the 244 patients, 40 individuals (16.4%) exhibited no functional limitations, categorized as grade 0 on the PCFS scale. Meanwhile, 100 patients (40.9%) experienced negligible functional limitations, and 14 patients (5.7%) faced severe functional limitations (Table 1).

**Table 1:** Comorbidities and drug history of the respondents (n = 244). COPD, chronic obstructive pulmonary disease; TB, tuberculosis

Comorbidities	Frequency (%)
Hypertension	92 (69.7)
Diabetes	64 (48.5)
Bronchial asthma	36 (27.3)
Heart disease	24 (18.2)
Rheumatoid arthritis or spondyloarthritis	6 (4.5)
Thyroid disease	6(4.5)
Heart failure	4 (3.0)
COPD	4 (3.0)
Cancer	2 (1.5)
Drug history	
Antihypertensive	86 (71.7)
Anti-diabetic	62 (51.7)
Asthma or COPD medication	36 (30)
Anti-stroke medication	2(1.7)
Anti-TB medication	2(1.7)
Cancer medication	2(1.7)

These post-COVID-19 complications significantly affect the patient’s overall well-being and functional status (Table 2). Fatigue was particularly prevalent, involving a large majority (*n *= 190, 77.9%) of the patients. Sleep disturbance was also common, with 126 (51.6%) patients reporting it. Anxiety (*n* = 102, 41.8%) and breathing difficulty (*n *= 98, 40.2%) were additional complications that substantially impacted the patients' lives. The study also identified psychological complications such as depression(*n *= 52, 21.3%), dementia-like syndrome (*n *= 36, 14.8%), and post-traumatic stress disorder (PTSD) (*n *= 36, 14.8%). Physical manifestations, including arthritis (*n *= 24, 9.8%), peripheral neuropathy (*n *= 16, 6.6%), seizures (*n *= 8, 3.3%), and bed sores (*n *= 6, 2.5%), further contributed to the burden experienced by these patients. Additionally, rare complications such as ischemic or hemorrhagic stroke (*n *= 4, 1.6%), anorexia (*n *= 4, 1.6%), dyspepsia (*n *= 4, 1.6%), deep vein thrombosis (DVT, *n *= 2, 0.8%), and skin rash or urticaria (*n *= 2, 0.8%).

**Table 2:** Association of the patients between post-COVID-19 functional status and post-COVID-19 complications (n = 244). **P < 0.05. ***P < 0.01.

Post-COVID-19 complications	Functional status in the PCFS scale, frequency (%) (within the column)					
	Total, *n* (%)	Grade 0, *n* (%)	Grade 1, *n* (%)	Grade 2, *n* (%)	Grade 3, *n* (%)	Grade 4, *n* (%)
Fatigue						
Yes	190 (77.9)	28 (70.0)	72 (72)	42 (91.3)	38 (86.4)	10 (71.4)
No	54 (22.1)	12 (30)	28 (28)	4(8.7)^**^	6 (13.6)	4 (28.6)
Sleep disturbance						
Yes	126 (51.6)	12 (30.0)	38 (38.0)	36 (78.3)	30 (68.2)	10 (71.4)
No	118 (48.4)	28 (70.0)^***^	62 (62.0)^***^	10 (21.7)^***^	14 (31.8)^**^	4 (28.6)
Anxiety						
Yes	102 (41.8)	12 (30.0)	32 (32.0)	22 (47.8)	28 (63.6)	8 (57.1)
No	142 (58.2)	28 (70.0)	68 (68.0)^***^	24 (52.2)	16 (36.4)^***^	6 (42.9)
Breathing difficulty						
Yes	98 (40.2)	4 (10)	30 (30)	18 (39.1)	34 (77.3)	12 (85.7)
No	146 (59.8)	36 (90)^***^	70 (70)^***^	28 (60.9)	10 (22.7)^***^	2 (14.3)^***^
Depression						
Yes	52 (21.3)	4 (10.0)	18 (18.0)	10 (21.7)	16 (36.4)	4 (28.6)
No	192 (78.7)	36 (90.0)	82 (82.0)	36 (78.3)	28 (63.6)^**^	10 (71.4)
Dementia-like syndrome						
Yes	36 (14.8)	6 (15.0)	16 (16.0)	8 (17.4)	2 (4.5)	4 (28.6)
No	208 (85.2)	34 (85.0)	84 (84.0)	38 (82.6)	42 (95.5)^**^	10 (71.4)
Post-traumatic stress disorder						
Yes	36 (14.8)	2 (5.0)	6 (6.0)	16 (34.8)	12 (27.3)	0 (0.0)
No	208 (85.2)	38 (95.0)	94 (94.0)^***^	30 (65.2)^***^	32 (72.7)^**^	14 (100.0)
Arthritis						
Yes	24 (9.8)	0 (0.0)	8 (8.0)	10 (21.7)	6 (13.6)	0 (0.0)
No	220 (90.2)	40 (100.0)^**^	92 (92.0)	36 (78.3)^***^	38 (86.4)	14 (100.0)
Peripheral neuropathy						
Yes	16 (6.6)	0 (0.0)	6 (6.0)	4 (8.7)	4 (9.1)	2 (14.3)
No	228 (93.4)	40 (100.0)	94 (94.0)	42 (91.3)	40 (90.9)	12 (85.7)
Personality change						
Yes	16 (6.6)	0 (0.0)	12 (12.0)	4 (8.7)	0 (0.0)	0 (0.0)
No	228 (93.4)	40 (100.0)	88 (88.0)^***^	42 (91.3)	44 (100.0)	14 (100.0)
Seizure						
Yes	8 (3.3)	2 (5.0)	4 (4.0)	0 (0.0)	2 (4.5)	0 (0.0)
No	236 (96.7)	38 (95.0)	96 (96.0)	46 (100.0)	42 (95.5)	14 (100.0)
Bedsore						
Yes	6 (2.5)	0 (0.0)	0 (0.0)	2 (4.3)	2 (4.5)	2 (14.3)
No	236 (96.7)	40 (100.0)	100 (100.0)	44 (95.7)	42 (95.5)	12 (85.7)
Ischemic or hemorrhagic stroke						
Yes	4 (1.6)	0 (0)	4 (4)	0 (0)	0 (0)	0 (0)
No	240 (98.4)	40 (100)	96 (96)^**^	46 (100)	44 (100)	14 (100)^**^
Anorexia						
Yes	4 (1.6)	2 (5.0)	0 (0.0)	2 (4.3)	0 (0.0)	0 (0.0)
No	240 (98.4)	38 (95.0)	100 (100.0)	44 (95.7)	44 (100)	14 (100)
Dyspepsia						
Yes	4 (1.6)	0 (0)	2 (2)	2 (4.3)	0 (0.0)	0 (0.0)
No	240 (98.4)	40 (100)	98 (98)	44 (95.7)	44 (100)	14 (100)
Deep vein thrombosis						
Yes	2 (0.8)	0 (0)	0 (0.0)	0 (0)	0 (0.0)	2 (14.3)
No	242 (99.2)	40 (100)	100 (100.0)	46 (100)	44 (100)	12 (85.7)^***^
Skin rash or urticaria						
Yes	2 (0.8)	0 (0)	0 (0.0)	2 (4.3)	0 (0.0)	0 (0.0)
No	242 (99.2)	40 (100)	100 (100.0)	44 (95.7)^**^	44 (100)	14 (100)

Post-COVID-19 complications have been found to significantly impact various aspects of functional status. Sleep disturbance and breathing difficulties were found to be notably associated with most grades of functional status. Sleep disturbance showed statistical significance with all grades except grade 4 (*P *≤ 0.05), while breathing difficulties were significantly associated with all grades except grade 2 (*P *≤0.05). Arthritis was found to have a statistical association with grade 0 (*P *≤ 0.05). On the other hand, anxiety, PTSD, personality change, and ischemic or hemorrhagic stroke were found to be significant with grade 1 (*P *≤ 0.01). Fatigue, PTSD, arthritis, and skin rash or urticaria showed significance with grade 2 (*P *≤ 0.05). All mental health-related complications, including depression, anxiety, PTSD, and dementia-like syndrome, were found to be statistically significant with grade 3 (*P *≤ 0.05). In severe functional limitation (grade 4) cases, DVT, and ischemic or hemorrhagic stroke showed statistical significance.

## Discussion

To the best of our knowledge, this is the first study in Bangladesh to evaluate persistent functional limitations among convalescent COVID-19 patients using the PCFS scale and to examine its association with post-COVID complications. Among the respondents, 54.1% had comorbidities, with hypertension being the most common (92, 69.7%), followed by diabetes (64, 48.5%), and bronchial asthma (36, 27.3%). These findings are consistent with previous studies, which have reported that extra-pulmonary multi-organ dysfunction and pre-existing conditions such as diabetes and cardiovascular diseases are significantly elevated in COVID-19 patients following discharge [11,12,13]. Fatigue, myalgia, arthralgia, and reduced physical activities were identified as the most common physical post-COVID complications, consistent with the current study findings [2,14].

To our knowledge, this study is the first to evaluate persistent functional limitations among convalescent COVID-19 patients in Bangladesh using the PCFS scale and explore its association with post-COVID complications. Fatigue was a prominent complication, affecting 77.9% of the participants, and was significantly associated with persistent limitations in functional activity across all PCFS grades. Sleep disturbance, reported by 126 (51.6%) respondents, also showed a significant association with functional limitations, particularly in higher PCFS grades. Other complications, such as anxiety, breathing difficulties, depression, dementia-like syndrome, PTSD, arthritis, peripheral neuropathy, personality change, seizures, bed sores, ischemic or hemorrhagic stroke, anorexia, dyspepsia, DVT, and skin rash or urticaria, demonstrated similar associations with functional status.

These findings suggest that various post-COVID-19 complications are associated with a higher likelihood of persistent functional limitations, with fatigue and sleep disturbance being particularly notable. Anxiety, breathing difficulties, and dementia-like syndrome also significantly impacted functional status. Conversely, conditions such as depression and peripheral neuropathy did not consistently associate with persistent functional limitations.

Previous studies have similarly explored the association between post-COVID-19 complications and functional status [5-9]. For instance, Pant et al. [15] conducted a study in Nepal on the prevalence of functional limitations in COVID-19 recovered patients using the post-COVID-19 functional status scale, finding fatigue, shortness of breath, and joint pain to be the most common persistent symptoms, which were associated with impaired ]functional status and reduced quality of life. Mohamed Hussein et al. [16] examined post-COVID-19 functional status regarding age, smoking, hospitalization, and previous comorbidities, finding that a substantial proportion of older adults experienced a decline in functional abilities even after recovery from acute illness. Another cross-sectional survey indicated that approximately one-third of respondents reported persistent symptoms, including fatigue, dyspnea, and impaired physical function, associated with decreased physical and mental health-related quality of life [17].

Recent studies have also highlighted the role of immune dysregulation and persistent inflammation in driving long-term symptoms. For instance, Phetsouphanh et al. [18] found that immune activation and cytokine dysregulation persist in long COVID patients, potentially explaining symptoms such as fatigue and cognitive impairment. Similarly, Su et al. [19] reported that endothelial dysfunction and microvascular damage could contribute to persistent respiratory and neurological symptoms in COVID-19 survivors. These are the plausible explanations for the persistent symptoms seen in COVID-19 survivors, such as fatigue, cognitive impairment, respiratory, and neurological issues.

Strengths 

Understanding the association between post-COVID-19 complications and functional status is crucial for identifying individuals who may benefit from targeted rehabilitation interventions. By recognizing the specific complications most strongly associated with persistent functional limitations, healthcare providers can tailor treatment plans and support strategies to address the unique needs of COVID-19 survivors.

Limitations

This study, however, has some limitations. It was conducted in a single institution, which might not represent the broader scenario in Bangladesh. Additionally, the sample size was relatively small, which may limit the generalizability of the findings. Future research should aim to include a broader and more diverse population to validate these findings and further explore the rehabilitation needs of COVID-19 survivors.

## Conclusions

A significant proportion of individuals who recovered from COVID-19 continue to experience a range of complications; among them, fatigue and sleep disturbances were the most prevalent complications, significantly impacting daily functioning and quality of life. The study highlights the need for targeted rehabilitation and multidisciplinary care to address these long-term consequences. Identifying specific post-COVID complications associated with functional limitations underscores the importance of tailored interventions to improve the overall well-being of COVID-19 survivors.

## Appendices

Appendix 

**Table 3:** Post-COVID-19 Functional Status Scale

How much are you currently affected in your everyday life by COVID-19?	Corresponding PCFS scale grade if the box is ticked if the box is ticked	
I have no limitations in my everyday life and no symptoms, pain, depression or anxiety.		0
I have negligible limitations in my everyday life as I can perform all usual duties/activities, although I still have persistent symptoms, pain, depression or anxiety.		1
I suffer from limitations in my everyday life as I occasionally need to avoid or reduce usual duties/activities or need to spread these over time due to symptoms, pain, depression or anxiety. I am, however, able to perform all activities without any assistance.		2
I suffer from limitations in my everyday life as I am not able to perform all usual duties/activities due to symptoms, pain, depression or anxiety. I am, however, able to take care of myself without any assistance.		3
I suffer from severe limitations in my everyday life: I am not able to take care of myself and therefore I am dependent on nursing care and/or assistance from another person due to symptoms, pain, depression or anxiety.		4
